# Is the temperament of crossbred dairy cows related to milk cortisol and oxytocin concentrations, milk yield, and quality?

**DOI:** 10.1371/journal.pone.0286466

**Published:** 2023-06-01

**Authors:** Maria Guilhermina Marçal-Pedroza, Mariana Magalhães Campos, Marta Fonseca Martins, Marcos Vinícius Barbosa Silva, Mateus José Rodrigues Paranhos da Costa, João Alberto Negrão, Aline Cristina Sant’Anna

**Affiliations:** 1 Nucleus of Studies and Research in Ethology and Animal Welfare (NEBEA), Department of Zoology, Institute of Biological Sciences, Federal University of Juiz de Fora, Juiz de Fora, Minas Gerais, Brazil; 2 Postgraduation Program in Biodiversity and Nature Conservancy, Federal University of Juiz de Fora, Juiz de Fora, Minas Gerais, Brazil; 3 Brazilian Agricultural Research Corporation, Embrapa Dairy Cattle, Minas Gerais, Brazil; 4 National Council for Scientific and Technological Development, CNPq Researcher, Brasília, Brazil; 5 Research Group in Ethology and Animal Ecology, Department of Animal Science, Faculty of Agriculture and Veterinary Sciences, São Paulo State University (UNESP), Jaboticabal, São Paulo, Brazil; 6 Basic Science Department, Faculty of Animal Science and Food Engineering (FZEA), São Paulo State University (USP), Pirassununga, São Paulo, Brazil; Universidade Federal de Mato Grosso do Sul, BRAZIL

## Abstract

Reactive dairy cows are more susceptible to stress, and this may result in negative effects on milk yield and quality. The aims of this study were to investigate the relationships between temperament traits and concentration of milk cortisol and oxytocin, milk yield, milkability, and milk quality in Holstein-Gyr cows. Temperament traits were assessed in 76 Holstein-Gyr cows in the milking parlor (by scoring milking reactivity and recording the numbers of steps and kicks during pre-milking udder preparation and when fitting the milking cluster) and during handling in the corral (by measuring the time to enter in the squeeze chute, ET and flight speed, FS). Milk samples were collected for milk quality (% fat, % protein, % lactose, and somatic cell count, SCC), and milk cortisol and oxytocin. Milk yield, milking time, and average flow were also measured. The calmer cows during milking management (class ‘low’) produced milk with higher protein (p = 0.028) content and tendencies for lower fat (p = 0.056) and higher lactose (p = 0.055) contents. Regarding the hormones, the most reactive cows (class ‘high’) in the milking and handling corral produced milk with higher concentrations of cortisol (p<0.001) and oxytocin (p = 0.023). In addition, the temperament of the animals affected some of the productive measures evaluated. Cows with reactive temperament had lower milk flow and longer milking time than the intermediate ones and had higher fat and a tendency for lower protein percentage in milk compared to cows with intermediate temperaments. Calm and intermediate cows in the handling corral produced more milk and presented better milkability parameters, such as a shorter milking time and greater average milk flow. Our results suggest that the cows’ behavioral reactivity can be related to the intensity of their response to stress during handling.

## 1. Introduction

Bovines, like other animals, present individual differences in behavior when exposed to challenging situations, and these behavioral differences are often described as temperament [1]. Temperament is expressed through a set of behavioral and physiological responses as a strategy to adapt to stressful situations in the environment [2]. However, most studies recognize that the characterization of temperament is complex since it can consider various traits, such as coping style, emotionality, and sociability [1, 3].

Studies have shown the importance of cattle temperament in livestock husbandry. Some studies have reported that calmer and more docile dairy cows in the milking parlor (milking temperament) produced greater milk yield [4–5], while others have found opposite results [6, 7] or did not find any association between milking temperament and milk yield [8, 9], showing a lack of consistency among results. It is important to highlight that these articles used different methods to assess milking temperament. Hedlund and Løvlie [10]; Marçal-Pedroza et al. [11]; and Neave et al. [5] used the number of steps and kicks as measures of reactivity during milking procedure. Breuer et al. [4]; Sutherland et al. [8]; Sutherland and Huddart [9] measured reactivity based on the intensity of leg movements, whereas Gergovska et al. [6, 12] and Sawa et al. [7] assigned subjective temperament scores.

Additionally, there is a lack of studies assessing the relationship between cows’ temperament, milk quality [12], and milkability parameters [13, 14]. Some of these studies have indicated that calmer animals produced milk with greater contents of fat and protein [15, 16], while others showed contrasting results, with the most reactive cows showing higher percentages of fat in the milk [17]. It has also been reported that calmer cows had better milkability parameters, such as greater milk flow and lower milking time [13, 14]. Considering the small number of studies addressing these issues and the divergent results, more research is needed to clarify the underlying behavioral and physiological factors affecting the relationship between temperament and productivity of dairy cows. All these cited studies used reactivity scores in the milking parlor to measure the milking temperament.

It is of particular interest to assess the temperament of dairy cattle breeds known for expressing a more reactive temperament, reacting more intensely and with greater agitation to the handling procedures [18]. Among them, we highlight the dairy Gyr cattle [19], which are widely used for crossbreeding in tropical countries, like Brazil, where around 80% of the dairy herd are Holstein-Gyr crossbred cows [20]. Under such conditions, it is expected that the crossed dairy cows with a greater Zebu breed composition will be more reactive to milking management, which may result in negative effects on milk yield and quality. Along with a higher cortisol concentration, a reduction in plasma oxytocin concentration is also expected [21], which is responsible for milk ejection and maintenance of lactation [22]. Few studies have investigated the relationship between oxytocin concentration and the temperament of dairy cows and they have found contradictory results. Sutherland and Tops [23] showed that Zebu crossbred cows displaying higher levels of agitation (measured by a reactivity score during the milking cluster attachment) in a new milking environment tended to present a greater concentration of blood oxytocin, but Sutherland et al. [10] did not find any association between reactivity in the milking parlor and the concentration of plasmatic oxytocin.

Thus, this study aimed to investigate the relationships between temperament traits and concentration of milk cortisol and oxytocin, milk yield, milkability, and milk quality in Holstein-Gyr cows. We hypothesized that more reactive cows in the milking parlor (with higher reactivity scores, more steps, and kicks) and in the handling corral (entered and exited the squeeze chute faster) would have higher concentrations of milk cortisol, oxytocin, and produce less milk with lower quality.

## 2. Material and methods

This study is in accordance with the ethical principles of animal experimentation and was approved by the Embrapa Dairy Cattle Animal Care and Use Committee, Juiz de Fora, MG, Brazil (Protocol n. 5201240417).

### 2.1. Animals and handling

The study was carried out in the Campo Experimental da Embrapa Gado de Leite ‘José Henrique Bruschi’ (Coronel Pacheco, MG), by evaluating 76 Holstein (H)-Gyr (G) primiparous and multiparous cows with 2.75 ± 1.35 lactations (mean ± SD), average daily milk yield 19.90 ± 6.30 kg, and days in lactation 138.56 ± 91.91 at the beginning of the study. The animals were classified in four breed compositions: ^3^/_8_HG (n = 8); ½HG = F1 HG (n = 25); ¾HG (n = 35) and ^7^/_8_HG (n = 8). Cows were kept on pasture and were milked twice a day in a herringbone milking parlor (2 × 6), beginning at 07.30 a.m. and 03.00 p.m., always by the same milker, who was previously trained in good handling practices.

### 2.2. Temperament assessment

The behavioral responses of all 76 animals were assessed during the handling routines in the milking parlor (milking temperament) and the corral (handling temperament). The milking temperament was assessed during the morning milking for three consecutive days per month from June to August 2018, resulting in nine repeated measurements per cow. Only one milker and one observer were present during the behavioral recordings. The milker prepared each cow individually to be milked, so the observer could record the behavior of each cow in a direct and individualized manner. The reactivity measurements were taken by only one previously trained observer, considering the movement of the hind legs based on the following criteria: a) Reactivity score which is a behavioural-based score of the type and intensity of leg movement, assessed during pre-milking udder preparation (RSprep, from the first contact of the milker with the cow’s teats, pre-dipping, evaluation of forestripping milk until the drying of teats) and when fitting the milking cluster (RStca, from the beginning of attachment of the first until the attachment of the last teat cup), by attributing one of the following scores: 1 = hind legs remained immobile throughout the procedure; 2 = one or two slow and gentle movements (hoof elevated at less than 15 cm from the ground) performed with one or both hind legs; 3 = three or more inconstant slow and gentle movements; 4 = constant (most of the observation time) slow and gentle movements; 5 = vigorous (elevating hooves above 15 cm from the ground), but inconstant movements; 6 = constant (most of the observation time) and vigorous movement of the hind limbs; 7 = the cow kick (elevating the hind hoof above hock line and directing it laterally towards the stockperson) and 8 = had to have one or both hind legs tied to be milked; b) Number of STEPS (elevations of the hooves below the hock line): corresponds to the sum of steps the animals took during pre-milking udder preparation and when fitting the milking cluster; c) Number of KICKS (defined as elevations of the hind hoof above hock line and directing it laterally towards the stockperson): corresponds to the sum of kicks during pre-milking udder preparation and during when fitting the milking cluster.

The handling temperament was assessed one day after assessing milking temperament, totalling three recordings throughout the study (one per month). The behavioral recordings were performed by individual observations for each animal by another observer who was unfamiliar with the animals and had experience with handling temperament assessment. Briefly, after the morning milking, the farm workers took the cows to a handling corral close to the milking parlor in a calm manner, according to the good management practices used on the farm. The following measurements were taken: a) Entrance time (ET), by measuring the time (in seconds) that each animal takes to go through the single-file race until entering the squeeze chute. The cow was allowed to move alone for ten seconds, without using any mechanism to encourage it to move. After this interval, those cows who stopped and refused to move forward were encouraged to move using voice command and, if necessary, were gently touched until they entered the squeeze chute [24]; and b) Flight speed (FS), by measuring the speed that each cow left the squeeze chute. It was done using equipment (Duboi^®^, Campo Grande, Brazil) comprised of two pairs of photoelectric cells and a chronometer, one of them fixed just after the exit gate of the squeeze chute and the other 2 m away. When the cow went through, the first pair of cells and the chronometer were activated, and were stopped when she went through the second pair. The time interval displayed on the equipment was used to calculate the speed of each cow, in m/s (faster animals were considered the most reactive ones).

### 2.3. Milk cortisol and oxytocin

The samples used to measure the concentrations of oxytocin and cortisol were collected during the morning milking, simultaneously with the milk collections for milk quality assessment, and on the last day of each milking temperament session (the third day of each monthly assessment). For the cortisol and oxytocin analyses, only ½HG and ¾HG cows were included to reduce the variation due to genetic composition. Among the 60 cows available (½HG, n = 25; ¾HG, n = 35), some had more than 6 lactations, or more than 180 days in lactation, or had clinical signs of mastitis on the days of milking sampling, and therefore were excluded. Thus, a subsample of 38 cows (½HG, n = 19 and ¾HG, n = 19) were assessed for these analyses. Hormones were measured in milk by immunoassay analysis (EIA) using commercial kits according to the manufacturer’s instructions (cortisol: Monobind, Lake Forest, CA, EUA; oxytocin: Mybiosource, San Diego, CA, EUA). As hormone concentrations in milk were substantially lower than those measured in plasma, we extracted milk samples. Briefly, we centrifuged the milk sample to separate the fatty and aqueous fractions. Each fraction was lyophilized, and the milk samples were 10-fold less diluted than the plasma samples. Regarding the milk, the intra-assay CVs were 4.8 and 6.5, and the inter-assay CV was 6.0 and 9.0% for cortisol and oxytocin, respectively.

### 2.4. Productive performance and milkability parameters

The individual daily milk production (kg/day), daily milking time (average of morning and afternoon milkings, in seconds), average milk flow (average of morning and afternoon milkings, in kg/s), and lactation days were manually recorded by the same observer who performed the behavioral observations, one day after performing the milking temperament assessment.

### 2.5. Milk quality indicators

To assess milk quality (percentage of fat, protein, lactose, and somatic cell count), individual milk samples were collected from all 76 cows, always on the last day of each of the three-monthly data collections in the milking parlor. The milk samples were kept in plastic containers of 50 mL each. The Centesimal Composition Analysis and Somatic Cell Count in Raw Milk Samples tests were performed at Embrapa Gado de Leite (Juiz de Fora, MG, Brazil). The analyses of fat, protein, and lactose content (% = g/100 g of raw milk) were carried out via absorption spectrometry in a mid-range infrared sensor (ISO 9622 | IDF 141) (Bentley Instruments, Bentley FTS, Id.: 85015); whereas the somatic cell count was performed via Flow cytometry (ISO 13366–2 | IDF 148–2); (Bentley Instruments, SomaCount FCM, Id.: 82015).

### 2.6. Data analysis

First, a descriptive statistical analysis of the data (S1 Data) from each evaluation month was carried out using the UNIVARIATE process of the SAS statistical package (SAS Inst. Inc. Cary. NC, version 9.3). Then, we used the Kolmogorov–Smirnov test to assess whether the distribution of milking temperament measures (RSprep, RStca, STEPS, and KICKS) and handling temperament measures (ET and FS), production and physiology variables met normality. We also checked if the temperament measures differed across the months and between the breed compositions, using linear mixed models for repeated measures, via PROC MIXED of SAS, including each temperament measurement as a dependent variable, and the fixed effects of breed composition (^3^/_8_HG, ½HG, ¾HG, and ^7^/_8_HG), month (1 to 3), parity (1, 2, 3, and 4 or more calvings) and the random effect of animal. The temperament measures did not differ between the months of evaluation (P > 0.05 for all). Regarding the breed composition, we found a significant effect for RSprep (p = 0.031) and FS (p = 0.002), with ^3^/_8_HG and ^1^/_2_HG cows being more reactive (higher averages for both traits) than the other breed compositions. Parity did not affect any of the temperament measures evaluated (P > 0.05 for all).

To assess the relations of milking temperament with cortisol and oxytocin concentrations, milk yield, milkability parameters, and milk quality parameters, first, we calculated the individual monthly averages of milking temperament measures (RSprep, RStca, numbers of STEPS and KICKS), milk yield, and milkability to eliminate the ‘day’ effect and obtain a single monthly measure for all of the measures studied (3 repetitions, from June to August). Then we categorize the temperament to include them as fixed effects in the models (classes low, intermediate, and high). The categorization was done based on the tertiles of distribution for the 76 cows within each month (the first tertile was categorized as ‘low’, the second as ‘intermediate’, and the third tertile as ‘high’ for each temperament measure). Considering the low occurrence of KICKS its distribution was considered binomial, so this variable was categorized as “low” = no occurrence of kicks and “high” = 1 or more occurrence of kicks. We did a chi-square test in contingency table to determine if there were differences in the temperament categories distribution between the three months. Non-significant results (P > 0.05) were obtained for all of the temperament measures, showing that the temperament categories distributions did not change across the months.

Finally, linear mixed models were fitted using PROC MIXED of SAS when the residuals attained normality and generalized linear models using PROC GLIMMIX for somatic cell count, adopting lognormal distribution of dependent variable. The models included as dependent variables the concentration of cortisol and oxytocin, average daily milk production (in kg/day), milkability parameters (milking time and milk flow), milk quality (percentages of fat, protein, and lactose, and somatic cell count), and the fixed effects of temperament measurements (one trait included at a time), assessment month (1 to 3), breed composition, parity and days in lactation as covariates with linear effect. In all models, the random animal effect (*SUBJECT*) was considered as a repeated measurement within the evaluation month (1 to 3). In all of the analyses *P-*values ≤ 0.05 were considered as significant and ≤ 0.10 were discussed as trends.

## 3. Results

### 3.1. Relationships between temperament and concentrations of milk cortisol and oxytocin

Milk cortisol was related to the milking temperament, assessed by RSprep (p<0.001), RStca (p<0.001), STEPS (p<0.001), and a tendency for KICKS (p = 0.087) (Table 1). Cows with a greater reactivity during pre-milking udder preparation (RSprep-_High_) had 95.05% more cortisol in their milk than calmer cows (RSprep-_Low_). Animals classified in the RStca-_High_ had a cortisol concentration 100.09% greater than the cows classified as RStca-_Low_. Cows that took more steps during the milking (STEPS-_High_) had 81.43% more cortisol in their milk than cows with a calm temperament (STEPS-_Low_). Finally, animals that kicked during milking tended to have 28.40% more cortisol in their milk when compared to cows that did not kick. Regarding handling temperament, cows in the FS-_Inter_ category tended to have 36.96% more cortisol than FS-_low_ individuals (p = 0.088). These results indicate that reactive cows had a higher concentration of cortisol in milk.

**Table 1:** Least-square means (± SE) of concentration of cortisol and oxytocin as a function of classes of temperament indicators (n = 38).

Dependent variables^1^	Temperament classes			*F*_2,104_	*P*-value
	Low	Intermediate	High		
	RSprep				
Cortisol, ng/ml	6.23 ± 0.56 ^b^	7.35 ± 0.54 ^b^	12.15 ± 1.12 ^a^	10.87	<0.001
Oxytocin, pg/ml	5.29 ± 0.49 ^b^	5.75 ± 0.47 ^b^	7.82 ± 0.99 ^a^	2.54	0.083
	RStca				
Cortisol, ng/ml	5.44 ± 0.60 ^b^	6.89 ± 0.54 ^b^	10.88 ± 0.71 ^a^	17.56	<0.001
Oxytocin, pg/ml	5.82 ± 0.55 ^a^^b^	4.91 ± 0.49 ^b^	7.21 ± 0.65 ^a^	3.91	0.023
	STEPS				
Cortisol, ng/ml	6.03 ± 0.53 ^b^	7.23 ± 0.63 ^b^	10.93 ± 0.88 ^a^	11.36	<0.001
Oxytocin, pg/ml	5.50 ± 0.50	6.56 ± 0.56	5.01 ± 0.79	1.52	0.225
	KICKS			F _1,105_	
Cortisol, ng/ml	7.06 ± 0.44 ^b^	-	9.06 ± 1.05 ^a^	2.99	0.087
Oxytocin, pg/ml	5.76 ± 0.36	-	5.87 ± 0.87	0.01	0.910
	FS (m/s)				
Cortisol, ng/ml	6.19 ± 0.69 ^b^	8.48 ± 0.70 ^a^	7.88 ± 0.85 ^a^^b^	2.49	0.088
Oxytocin, pg/ml	4.74 ± 0.57 ^b^	6.49 ± 0.60 ^a^	6.50 ± 0.70 ^a^^b^	2.41	0.095
	ET (s)				
Cortisol, ng/ml	7.22 ± 0.83	7.16 ± 0.55	8.05 ± 0.85	0.40	0.673
Oxytocin, pg/ml	5.39 ± 0.68	5.74 ± 0.45	6.23 ± 0.70	0.36	0.699

^1^RSprep = reactivity scores during pre-milking udder preparation, RStca = reactivity scores when fitting the milking cluster, STEPS = number of steps, KICKS = number of kicks, ET = entrance time, FS = flight speed.

^a–b^ Means followed by the same letters in the row are not statistically different (P < 0.05).

The milking temperament was also related to oxytocin concentration, with significant effects for RStca (p = 0.023) and tendencies for the RSprep (p = 0.083) and FS (p = 0.095) measurements. The RSprep-_High_ cows had 49.5% more oxytocin in milk than RSprep-_Low_ cows (Table 1). The RStca-_High_ cows had 46.9% more oxytocin in milk than RStca-_Inter_ ones. Finally, milk from the animals in the FS-_High_ category had 36.83% more oxytocin than milk from cows in the FS-_Low_ category (Table 1). The ET was not related to milk cortisol and oxytocin concentrations (P > 0.05).

### 3.2. Relationships of temperament with milk yield and milkability

The milking temperament was not related to milk yield, or the milkability parameters (Table 2). Regarding handling temperament, ET had a significant relationship with milk yield (p = 0.004). Cows classified in the ET-_Inter_ category produced 27.62% more milk than ET-_High_ cows (Table 2). Among the milkability parameters, milking time was influenced by ET (p<0.0001) and FS (p = 0.0002). Cows with both extreme temperaments (high and low) for ET and FS were more difficult to milk and took more time to be milked than the intermediate ones. Cows classified as ET-_High_ spent 20.22% longer time being milked than ET-_Low_ cows. The same happened for animals who left the squeeze chute more slowly (FS-_Low_), which spent 19.91% longer being milked than FS-_High_ cows (Table 2). ET had also a significant relationship (p = 0.046) with milking flow. The ET-_Inter_ cows had a flow rate 14.80% faster than the ET-_Low_ cows, which did not significantly differ from ET-_High_.

**Table 2:** Least-square means (± SE) of milk yield and milkability traits as a function of the temperament indicators (n = 76).

Dependent variables^1^	Temperament classes			*F*_2,211_	*P*-value
	Low	Intermediate	High		
	RSprep				
Milk yield, kg/d	20.10 ± 1.23	18.67 ± 1.39	19.25 ± 1.50	0.57	0.565
Milking time, s	420.81 ± 12.83	435.80 ± 14.45	465.14 ± 18.15	2.22	0.111
Flow, g/s	20.45 ± 1.27	18.80 ± 1.46	21.67 ± 1.56	1.36	0.259
	RStca				
Milk yield, kg/d	19.62 ± 1.24	19.19 ± 1.36	19.56 ± 1.39	0.05	0.951
Milking time, s	421.16 ± 14.08	439.44 ± 14.04	450.36 ± 16.22	1.09	0.337
Flow, g/s	20.87 ± 1.29	19.67 ± 1.41	20.43 ± 1.45	0.33	0.718
	STEPS				
Milk yield, kg/d	20.55 ± 1.20	18.69 ± 1.44	18.43 ± 1.35	1.31	0.273
Milking time, s	435.72 ± 13.33	439.37 ± 15.53	431.19 ± 15.65	0.08	0.921
Flow, g/s	21.21 ± 1.25	18.88 ± 1.49	20.31 ± 1.41	1.20	0.303
	KICKS				
Milk yield, kg/d	19.08 ± 1.06	-	20.90 ± 1.61	F_1,211_ = 1.25	0.264
Milking time, s	432.91 ± 10.38	-	446.85 ± 19.50	F_1,210_ = 0.46	0.497
Flow, g/s	19.95 ± 1.10	-	22.15 ± 1.71	1.63	0.203
	FS (m/s)				
Milk yield, kg/d	21.05 ± 1.52	18.79 ± 1.12	19.69 ± 1.65	1.03	0.360
Milking time, s	516.44 ± 19.42 ^a^	435.89 ± 14.22 ^b^	430.68 ± 21.07 ^b^	8.77	0.0002
Flow, g/s	20.78 ± 1.61	20.00 ± 1.16	21.75 ± 1.74	0.58	0.562
	ET (s)				
Milk yield, kg/d	18.49 ± 1.18 ^b^	21.77 ± 1.25 ^a^	17.06 ± 1.71 ^b^	5.78	0.004
Milking time, s	416.38 ± 15.30 ^b^	494.35 ± 16.20 ^a^	500.60 ± 21.92 ^a^	10.34	<0.001
Flow, g/s	19.31 ± 1.24 ^b^	22.18 ± 1.31 ^a^	18.86 ± 1.79 ^a^^b^	3.13	0.046

^1^ RSprep = reactivity score during pre-milking udder preparation, RStca = reactivity score when fitting the milking cluster, STEPS = number of steps, KICKS = number of kicks, ET = entrance time, FS = flight speed.

^a–b^ Means followed by the same letters in the row are not statistically different (P < 0.05).

### 3.3. Relationship between milk temperament and milk quality

The milking temperament measured by RStca showed a tendency in the percentage of fat (p = 0.056). The milk from cows categorized as RStca-_Inter_ had 11.83% higher fat content than the milk from cows with lower reactivity (RStca-_Low_) (Table 3).

**Table 3:** Least-square means (± SE) of milk quality traits as a function of the temperament indicators (n = 76).

Dependent variables^1^	Temperament classes			*F*_*2*,*203*_	*P-value*
	Low	Intermediate	High		
	RSprep				
Fat, %	1.12 ± 0.05	1.15 ± 0.05	1.26 ± 0.06	2.07	0.129
Protein, %	3.33 ± 0.05 ^a^	3.33 ± 0.05 ^a^	3.17 ± 0.06 ^b^	3.63	0.028
Lactose, %	4.49 ± 0.06	4.47 ± 0.06	4.44 ± 0.07	0.20	0.817
SCC, log cel/ml	5.53 ± 0.20	5.16 ± 0.23	5.30 ± 0.25	1.40	0.249
	RStca				
Fat, %	1.12 ± 0.05 ^b^	1.25 ± 0.05 ^a^	1.19 ± 0.05 ^a^^b^	2.92	0.056
Protein, %	3.27 ± 0.05	3.30 ± 0.05	3.27 ± 0.05	0.19	0.825
Lactose, %	4.48 ± 0.06	4.43 ± 0.06	4.49 ± 0.06	0.53	0.588
SCC, log cel/ml	5.38 ± 0.20	5.52 ± 0.23	5.22 ± 0.23	0.74	0.478
	STEPS				
Fat, %	1.13 ± 0.05	1.24 ± 0.05	1.18 ± 0.05	1.99	0.140
Protein, %	3.31 ± 0.05 ^a^	3.19 ± 0.05 ^b^	3.30 ± 0.05 ^a^	2.46	0.088
Lactose, %	4.47 ± 0.05	4.42 ± 0.06	4.50 ± 0.06	0.70	0.498
SCC, log cel/ml	5.46 ± 0.20	5.44 ± 0.24	5.18 ± 0.23	0.73	0.481
	KICKS				
Fat, %	1.18 ± 0.04	-	1.14 ± 0.06	F_1,211_ = 0.33	0.568
Protein, %	3.26 ± 0.04	-	3.35 ± 0.06	F_1,211_ = 1.80	0.181
Lactose, %	4.46 ± 0.05	-	4.50 ± 0.07	F_1,208_ = 0.33	0.565
SCC, log cel/ml	5.42 ± 0.18	-	5.20 ± 0.27	F_2,213_ = 0.68	0.409
	FS (m/s)				
Fat, %	1.25 ± 0.06	1.14 ± 0.04	1.19 ± 0.06	1.86	0.158
Protein, %	3.23 ± 0.06	3.27 ± 0.04	3.32 ± 0.06	0.35	0.701
Lactose, %	4.56 ± 0.07	4.44 ± 0.05	4.43 ± 0.07	1.69	0.187
SCC, log cel/ml	5.21 ± 0.25	5.42 ± 0.19	5.45 ± 0.28	0.37	0.691
	ET (s)				
Fat, %	1.19 ± 0.05	1.12 ± 0.05	1.23 ± 0.07	1.98	0.140
Protein, %	3.33 ± 0.05 ^a^	3.25 ± 0.05 ^a^^b^	3.16 ± 0.07 ^b^	2.66	0.073
Lactose, %	4.41 ± 0.05 ^b^	4.55 ± 0.06 ^a^	4.44 ± 0.08 ^a^^b^	2.93	0.055
SCC, log cel/ml	5.45 ± 0.20	5.27 ± 0.21	5.33 ± 0.29	0.30	0.741

^1^RSprep = reactivity score during preparation for milking, RStca = reactivity score during milking cluster attachment, STEPS = number of steps, KICKS = number of kicks, ET = entrance time, FS = flight speed, SCC, somatic cell count.

^a–b^ Means followed by the same letters in the row are not statistically different (P < 0.05).

Regarding protein, cows with lower reactivity scores (RSprep-_Low_) produced milk with 5.21% higher protein content (p = 0.028) than the milk produced by cows of a more reactive temperament (RSprep-_High_). The cows classified as STEPS-_Inter_ tended (p = 0.088) to produce milk with 3.45% lower protein content when compared to cows classified as STEPS-_Low_ (Table 3). Protein content was also influenced by handling temperament, as the milk from cows with ET-_Low_ tended (p = 0.073) to have 5.24% greater protein content than the milk from cows with ET-_High_.

Lactose content tended to be related with ET (p = 0.055), as the milk from cows classified in the ET-_Inter_ category had 3.17% more lactose than cows with ET-_Low_ (Table 3). Finally, the SCC was not related to any of the temperament traits, either during milking or in the handling in the corral (Table 3).

## 4. Discussion

### 4.1. Relationships between temperament and concentrations of milk cortisol and oxytocin

The concentration of milk cortisol was greater for cows with a more reactive temperament during milking, as measured by our high reactivity scores during preparation and teat cup attachment, and by the high number of steps and tended to kick more during milking. It should indicate that these cows presented behavioral and physiological signs of stress during milking, suggesting that reactive cows are more susceptible to stress during routine handlings. This is similar to the findings by Wenzel et al. [25] and Gygax et al. [26] in which cows that kicked more or took more steps in the milking parlor produced milk with higher concentrations of cortisol when compared to their calmer counterparts. However, this differed from the results by Sutherland et al. [10] and Sutherland and Huddart [11], who evaluated the reactivity of the animals using reactivity scores similar to ours and did not find an association between the agitation of the cows in the milking parlor and the concentration of plasmatic cortisol. The same was reported by Van Reenen et al. [27], who did not find an association between the number of steps and kicks in milking and the concentration of plasmatic cortisol. These different results could be due to the cortisol sampling methods. In our study, we assessed the concentration of cortisol in the milk, as it is a less invasive method that does not cause additional stress during sampling collection. Van Reenen et al. [27]; Sutherland et al. [10] and Sutherland and Huddart [11] used blood sampling, which could increase the levels of plasmatic cortisol even in less reactive cows.

Blood cortisol is widely used to assess the neuroendocrine stress response [10, 11, 27, 28], but it is an invasive technique that could activate the HPA axis and cause an increase in plasma cortisol levels in cows [29]. A non-invasive alternative has been to measure cortisol in the milk. Cortisol, like other steroid hormones, can permeate and cross the epithelial layer between blood vessels and the alveoli of the mammary gland [29], resulting in a positive correlation between the concentration of cortisol in the blood and milk in response to different milking techniques [26, 30, 31]. Milk cortisol may be used as a biomarker to assess stress response to short- medium-term (12 h) environmental challenges in dairy cow [32].

Studies using ACTH challenge to investigate the changes in milk cortisol concentration found that the cortisol in milk might remain elevated until 8–10 h after receiving the stimulus, depending on the ACTH dosage [30, 31, 33]. In the study of Sgorlon et al. [34], the animals were milked twice a day (12 h intervals), as in the present study. In these situations, the cortisol concentration in the milk possibly reflects the variation of the plasma concentration in the interval of 10 to 14 h before the milk sampling, *i*.*e*. the previous milking [34].

Our results confirm the hypothesis that cows that are more reactive during milking are also more susceptible to physiological stress during handling and show a higher concentration of cortisol in milk. The high concentrations of cortisol and noradrenaline in the blood are associated with stress in the milking environment [35], as cortisol is one of the main hormones associated with physiological stress response in mammals [36]. A greater increase of this glucocorticoid occurs due to a stronger activation of the hypothalamic-pituitary-adrenal (HPA) axis in response to a stressing agent, that might be physical or emotional [36]. Individual differences in response to environmental stimuli are expected, and the variation in the glucocorticoid concentration has been associated with differences in temperament in beef cattle measured by the flight speed test [37].

The concentration of oxytocin was also higher in cows that presented greater reactivity scores during milking, as measured by high reactivity scores during teat cup attachment. Our results corroborate those of Sutherland and Tops [23], where cows with greater levels of RStca agitation in a new milking environment (psychological stressor) tended to present a greater concentration of blood oxytocin, suggesting that oxytocin may be related to the behavioral stress response in dairy cows. According to the authors, cows that present a heightened response to a psychological stressor and have higher concentrations of oxytocin could have greater stress coping mechanisms. In turn, Sutherland et al. [10] did not report any association between reactivity in a familiar milking parlor and concentrations of plasmatic oxytocin.

Oxytocin is the hormone responsible for milk ejection and maintenance of lactation [22], but has also been pointed to as a physiological reaction to stressing agents [10, 23]. In our study, the milk from reactive cows had higher cortisol and oxytocin concentrations, suggesting that a higher concentration of oxytocin might be part of the stress response in these cows, likely as a stress coping mechanism. That may occur as an attempt to mitigate the effects of stress during the milking process, as oxytocin has anti-stress [38] and anxiolytic effects [39], both associated with the HPA axis [37, 38]. However, some studies report that a high oxytocin concentration in female rodents leads to a decrease in cortisol concentration [39]. The same happens in dairy cows habituating to a new milking environment, where there is an increase in oxytocin release as the cows get used to the new environment [23], accompanied by a decrease in cortisol concentration. Sutherland et al. [10] found that in a new milking environment (psychological stressor), the blood cortisol concentration was greater before milking, and the oxytocin concentration was greater after milking. These results suggest that the level of cortisol before milking attenuated the oxytocin response to the new situation.

However, other studies have indicated that high levels of cortisol do not suppress the secretion of oxytocin [21, 40], similar to what occurred with the concentration of both hormones in the milk of our cows. Therefore, our results show that Holstein-Gyr crossbred cows with high reactivity had behavioral and physiological signs of stress during milking, even if they were milked in a familiar environment and by milkers using good handling practices, but the stress experienced by the cows seems not to affect the milk production. Reactive cows during milking had lower milk flow and longer milking time. They also showed an increase in oxytocin concentration during milking. Thus, a higher concentration of oxytocin does not necessarily mean a good milk ejection. That is, cows could release oxytocin and retain milk. Therefore, to analyze milking quality as a function of cows’ temperament, it is necessary to gather data from oxytocin release, milk flow, milking time, and milk yield.

Unlike milking temperament, the cows with intermediate handling temperament measured by FS tended to have higher concentrations of milk cortisol and oxytocin compared to those with extreme temperaments (low and high). These results differ from those of Sutherland et al. [10], who found that the more reactive cows (with high FS) had a higher basal cortisol concentration in a familiar milking environment (*i*.*e*. a rotary milking parlor where the cows were usually milked), but there was no variation in the cortisol concentration between cows of different FS categories exposed to an exogenous ACTH challenge. When exposed to a novel milking environment (a herringbone parlor within the same farm), these cows did not show variation in the concentration of plasmatic cortisol in relation to FS. In the same study, Sutherland et al. [10], working with multiparous cows, found that the concentration of blood oxytocin was higher for cows in the novel environment, regardless of FS category. However, in primiparous cows, the concentration of plasmatic cortisol was higher in cows with high FS during the first milking sessions [11]. In general, the authors found that the heifers previously trained to be milked reached lower plasmatic cortisol concentration. Flight speed is commonly used to assess differences in temperament for beef cattle [37, 41], but fewer studies have used this indicator for dairy cattle [6, 10, 42]. Since the concentration of cortisol and oxytocin had a positive and linear relationship with the reactivity measures during milking (but non-linear relation with the reactivity to handling in the corral), we might infer that the cows had different perceptions of the stimuli in the two distinct handling locations and reacted distinctively, resulting in different patterns of relationships between behavioral and physiological responses.

### 4.2. Relationships between temperament, milk yield, and milkability

We hypothesized that milking temperament would be related to milk yield based on previous studies reporting that cows who are more reactive to milking (measured by the number of steps and kicks) produced less milk [4, 5, 7]. Nevertheless, none of the milking temperament measures assessed in the present study were related to milk yield. The lack of association between milking temperament and milk yield was previously reported by Van Reenen et al. [27]; Orbán et al. [43]; and Sutherland and Huddart [11].

In contrast to the results reported by Sutherland and Dowling [44], Sutherland and Huddart [11], we did not find any association between FS and milk yield. Regarding milkability parameters, FS was associated with milking time and average milk flow. The cows which exited the squeeze chute slowly, considered to have a calmer temperament, spent more time being milked than more reactive cows, contrary to what we expected, but similar to what was reported by Sutherland and Huddart [11].

Among the handling temperament measures assessed in this study, only ET was related to milk yield, with cows classified as intermediate producing more milk than those classified as low and high for ET. It is possible that among the cows with the highest values for ET, some refused to walk and need to be stimulated with voice commands and / or touch to go into the squeeze chute. In its turn, those with the lowest ET values should include cows that entered running (i.e., more reactive ones). In this specific case, the Intermediate class should include animals with a better temperament that entered walking the single-file race and did not need to be stimulated to walk. Both extremes (low and high) for this measure, could be regarded as undesirable behaviors in the production environment. The ET was also related to milkability parameters since the intermediate cows showed greater average flow than the low and high classes. Furthermore, cows that took longer to enter the squeeze chute (possibly including cows that refused to walk as a response to fear), were the ones that took longer to be milked. Contrasting results were reported by Sutherland et al. [10], who found that dairy cows of intermediate temperament (average exit time–i.e., between 2 and 4s) reached a lower average flow when compared to those of calmer (exit time > 4s) and more reactive (exit time < 2s) temperaments, revealing a lack of consensus, that is probably related to the different types of temperament measures used.

It is interesting to highlight that few studies [11, 44, the present] evaluated the relationships between handling temperament with productive parameters for lactating dairy cows. Most of the studies with dairy cows limited the temperament assessment to the milking reactivity. In future studies, assessing the temperament of dairy cows should include indicators from different handling situations (beyond the milking parlor) to evaluate if the temperament in a broader sense could be related to productive parameters.

### 4.3. Relationship between temperament and milk quality

Calmer cows, measured by reactivity score during preparation, produced milk with a higher protein content and calmer cows during teat cup attachment tended to produce lower fat content. Similar results were found by Morales Pineyrúa et al. [45] for Holstein cows, in which calmer cows based on a milking reactivity score similar to ours, had lower protein and fat content. The handling temperament also influenced the milk quality. Cows that entered the squeeze chute faster (i.e., low class for ET) tended to have higher protein content while cows that entered the chute calmly (intermediate ET) tended to produced milk with higher contents of lactose than the faster cows. Kruszynski et al. [16] found that calmer cows produced milk with higher protein and fat contents. In turn, Cziszter et al. [17] reported that the milk produced by more agitated cows in the milking parlor had greater fat percentages than the milk from cows of intermediate temperament, which had a lower content of protein than the calmer and more agitated ones. In contrast, Gergovska et al. [12] found that both more agitated and calmer cows produced milk with a higher fat content than those of intermediate temperament. Finally, Orbán et al. [43] failed to find a significant effect of temperament on the protein and fat contents in the milk of Jersey and Holstein cows. All of these studies assessed temperament based on the cows’ reactivity during milking. The lack of consensus on the effect of dairy cows’ temperament on fat and protein milk contents is likely due to differences in temperament assessment methods, breed, or handling conditions. In the present study, animals with a calmer temperament in the milking parlor produced milk that could be regarded as more desirable by consumers of fluid milk, that is, with higher protein content and lower fat content [46]. The relationship between temperament and milk quality should be further investigated in future research since there are few studies published on this topic.

Finally, the present study had some limitations that must be discussed. The research was conducted on an experimental farm where the animals are handled more frequently, which would make them more habituated to handling (being regarded as ‘calmer’) than the average Zebu cows in Brazilian commercial herds. Additionally, our sample varied in days in lactation, parity, and genetic group. To standardize these sources of variation we would have to exclude animals from our sample, leading to an even lower sample size. Therefore, we decided to include all of the cows available in the herd and control for these factors in the statistical analyses. Finally, we expected to find a genetic group effect in the temperament measures, but we were not able to investigate this relationship because of the low sample of animals within each genetic group. Future studies on this topic should include larger samples of crossed Zebu cows to allow for the assessment of genetic group effects on temperament and hormone concentration. It would also be of interest to integrate physiological and temperament indicators assessed in different handling situations (corral and milking parlor) [3]. The inclusion of other tests traditionally used to assess temperament in cattle should also be investigated in future studies, such as novel object, novel human, avoidance distance, and restraint tests [7]. It would allow for a broader view of the cows’ temperament, including traits that go beyond milking reactivity. The integration of various temperament tests should be assessed using statistical methods for data dimensionality reduction, such as principal component analyses or factor analysis, which would help identify key components or factors that provide a better overall understanding of Zebu cows’ temperament.

## 5. Conclusions

We conclude that handling temperament is related to milk yield and milkability, since calm and intermediate cows in the handling corral produced more milk and presented better milkability parameters, such as a shorter milking time and greater average milk flow. Additionally, the cows with better temperament in the milking parlor (calm and intermediate cows) produced milk with lower fat content and higher protein content. More reactive cows during milking produced milk with higher concentrations of cortisol and oxytocin, showing that behavioral reactivity could be related to the intensity of the physiological stress response. Future studies should investigate measures that lead to the improvement of temperament of crossbred Zebu cows, such as genetic selection and the use of good practices of handling, with the aim of reducing the cows’ reactivity to handling and improving the welfare of the cows, the workers, and the productive indices, making the dairy industry more sustainable and efficient.

## Supporting information

S1 Data Data set used in the statistical analysis. (XLSX)
